# A Plan for the Disposal of Tuberculous Animals

**Published:** 1902-01

**Authors:** 


### A PLAN FOR THE DISPOSAL OF TUBERCULOUS ANIMALS.

We print in this issue a paper by "Columella," on a proposal of a new plan to combat tuberculosis of cattle conservatively. So far as we know, the proposal is original, and has not heretofore been advanced. The tuberculosis problem has been discussed at great length, but it remains the *bête noir* of the stockman and of the veterinarian. While we may at times tire of the discussion of this subject, it is necessary for us to continue to study and discuss it until a satisfactory solution shall be reached. If veterinarians do not settle this question, who shall?

While we approve of the plan in operation in Pennsylvania, we agree with "Columella" that it is not supported by a sufficient appropriation to cover the whole State adequately; and that if a cheaper method can be discovered—one that will permit more herds to be cleaned up with the present annual appropriation of $40,000—it will do much to hasten the eradication of this destructive disease. Veterinarians interested in the study of this great problem in cattle breeding, economics, and public health are invited to make use of these columns in discussing "Columella's" proposal. We should like to organize a symposium on this theme, in the hope that it may contribute to the solution of this greatest of veterinary problems.

In order that the symposium may be effective it is desired that the writers contributing to it shall adhere closely to the discussion of "Columella's" proposal and not digress.

Now, gentlemen, the tourney is called. Who will enter the lists?

